# Gateway-Assisted Retransmission for Lightweight and Reliable IoT Communications

**DOI:** 10.3390/s16101560

**Published:** 2016-09-22

**Authors:** Hui-Ling Chang, Cheng-Gang Wang, Mong-Ting Wu, Meng-Hsun Tsai, Chia-Ying Lin

**Affiliations:** Department of Computer Science and Information Engineering, National Cheng Kung University, Tainan 701, Taiwan; momo@imslab.csie.ncku.edu.tw (H.-L.C.); wjungle@gmail.com (C.-G.W.); aj211y@imslab.csie.ncku.edu.tw (M.-T.W.); a711186@imslab.csie.ncku.edu.tw (C.-Y.L.)

**Keywords:** Constrained Application Protocol (CoAP), Internet of Things (IoT), Message Queuing Telemetry Transport for Sensor Networks (MQTT-SN), retransmission mechanism, retransmission timeout (RTO), round-trip time (RTT)

## Abstract

Message Queuing Telemetry Transport for Sensor Networks (MQTT-SN) and Constrained Application Protocol (CoAP) are two protocols supporting publish/subscribe models for IoT devices to publish messages to interested subscribers. Retransmission mechanisms are introduced to compensate for the lack of data reliability. If the device does not receive the acknowledgement (ACK) before retransmission timeout (RTO) expires, the device will retransmit data. Setting an appropriate RTO is important because the delay may be large or retransmission may be too frequent when the RTO is inappropriate. We propose a Gateway-assisted CoAP (GaCoAP) to dynamically compute RTO for devices. Simulation models are proposed to investigate the performance of GaCoAP compared with four other methods. The experiment results show that GaCoAP is more suitable for IoT devices.

## 1. Introduction

The Internet of Things (IoT) is a network linking physical objects that generally have embedded sensors/actuators (SA) to sense events and interact with the environment. Those physical objects usually called devices communicate with each other with limited human interaction. When the devices detect some event happening, they generate data and send it to relative receivers. For example, the implantable medical devices are introduced to collect patients’ data (e.g., blood pressure or body temperature) [1]. Once the devices sense the change of patients’ status, the devices notify relevant medical units immediately.

**Figure 1:**
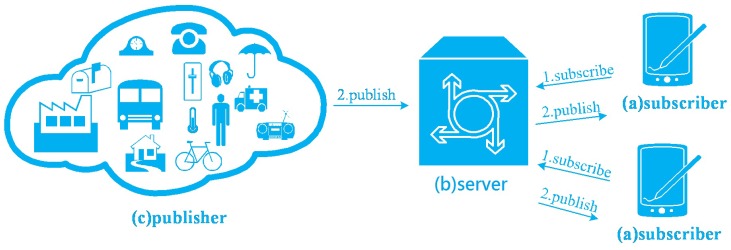
Publish/subscribe model.

There are two major messaging patterns: request/response and publish/subscribe. Request/response pattern is widely used in the Internet where data are stored in servers. The data transmission occurs because of users’ requests. This pattern is suitable for user-centric applications such as web browsing, since the data retrieval is passive. However, IoT applications are data-centric applications. Publish/subscribe patterns are widely used in IoT since IoT devices can sleep most of the time and wake up to publish data occasionally [2]. Figure 1 shows the simple publish/subscribe model.

The interested party called a subscriber registers their interest to the server. This registration process is called subscription (see step 1 in Figure 1). The publisher produces information and the server forwards the information from the publisher to the subscribers (see step 2 in Figure 1) [3].

This publish/subscribe model for IoT is supported by protocols such as Message Queuing Telemetry Transport for Sensor Networks (MQTT-SN) [4] and the Constrained Application Protocol (CoAP) [5]. For the purpose of staying lightweight, the transport layer of these two protocols are both User Datagram Protocols (UDPs). However, reliability of data transmission is one of the most important issues in some IoT applications with sensors, such as environmental condition monitoring [6]. In these applications, sensors have diverse reliability requirements. For example, temperature information in normal range can tolerate loss up to a certain percentage. On the other hand, the sensor data reflecting a high temperature should be reliably delivered to the control center. As a result, both MQTT-SN and CoAP provide retransmission mechanisms.

There is a timer called retransmission timeout (RTO). If the device does not receive the acknowledgement (ACK) before the RTO expires, the device will retransmit the data. RTO setting can be fixed or dynamic. For dynamic settings, the Round Trip Times (RTTs, from the devices to the server) measured from previous messages by the devices are used for computing RTO.

In unstable wireless environments, the performance of fixed RTO is poor. If the fixed RTO is too small, unnecessary retransmission occurs. While the fixed RTO is too large, the latency becomes quite large since the duration of each retransmission becomes long. Note that MQTT-SN and CoAP both apply fixed RTO. Thus, dynamic RTO can adapt to the wireless network and overcome the two problems above.

The network of IoT devices is usually connected using wireless with high data loss rate and unstable RTT. The existing protocols need to be modified to adapt to the network conditions. Moreover, the characteristics of IoT devices have to be considered. Our aim is to propose a gateway-assisted retransmission mechanism suitable for IoT devices to obtain dynamic RTO.

## 2. Related Works

In this section, we first introduce two popular lightweight publish/subscribe protocols that can be used in IoT. To minimize power consumption, both protocols adopt fixed RTO as retransmission criteria. We then describe two mechanisms using dynamic RTO.

**Figure 2:**
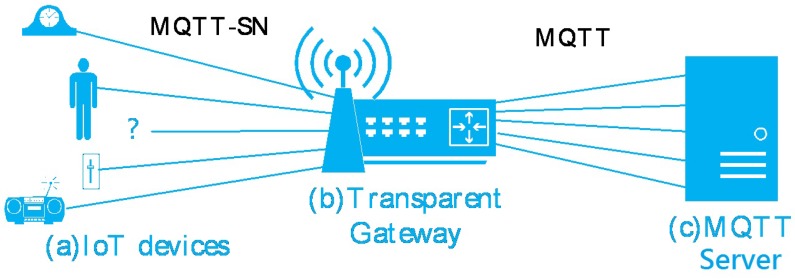
MQTT-SN architecture.

Message Queuing Telemetry Transport (MQTT) is a machine-to-machine connectivity protocol that runs over Transmission Control Protocol (TCP) [7]. It is designed for short length packets. MQTT-SN is a modified version of MQTT for sensor networks. Instead of TCP, MQTT-SN runs over UDP for lightweight purposes [4]. Figure 2 shows the architecture of MQTT-SN. Although IoT devices use the MQTT-SN protocol, they need a gateway to connect to the MQTT server while the connection between the gateway and the server is still the MQTT protocol. There are two types of MQTT-SN gateways: transparent gateways and aggregating gateways. Transparent gateways maintain each connection from the devices to the server but aggregating gateways only maintain one MQTT connection to each server. There are three levels of Quality of Service (QoS) that can be used when the data are delivered. Level 0 is called at-most-once delivery. This is best-effort delivery without any retransmission service so the data are always transmitted at most once. Level 1 is called at-least-once delivery. The data are delivered at least once. The device stores the data until it receives an ACK (called PUBACK) from the receiver. Level 2 is called exactly-once delivery, which guarantees that the data are received only once [4]. For QoS levels 1 and 2, a retransmission mechanism is required. MQTT-SN specification provides two parameters for retransmission: $T_{retry}$ and $N_{retry}$. The first one indicates a fixed RTO value recommended between 10 and 15 s. The second one represents the maximum number of retransmissions recommended from three to five times.

CoAP is proposed by the Internet Engineering Task Force (IETF) for constrained devices to connect to the Internet. Based on UDP, CoAP supports both reliable (called Confirmable or CON) and unreliable (called Non-Confirmable or NON) transmission. By using NON, there is no need to return ACK and no retransmissions occur. On the other hand, when the RTO of a device expires, the publication data will be retransmitted by using CON. *Initial RTO* is defined as the RTO used for the first transmission of a message. In CoAP, the default maximum number of retransmissions is four, and the initial RTO is randomly chosen from 2 to 3 s. Once the RTO expires before ACK reception, the next RTO is updated with exponential back-off mechanism. (i.e., if the initial RTO is two, the next RTO is updated to four and the third time will be to eight.) [5] Note that configuration of these parameters is application-specific. Without loss of generality, we only consider the default values proposed by RFC 7252 in this paper.

MQTT-SN and CoAP use fixed RTO, which is simple but may not work well in wireless networks. The dynamic RTO in TCP is defined in RFC 6298 [8]. The notations used in calculating dynamic RTO are shown in Table 1.

The following five rules of RTO are defined in RFC 6298:

1. When there is no RTT sample, the RTO is set to 1 (i.e., Ω=1); otherwise, the RTO is set based on smoothed round-trip time (SRTT, denoted as $\gamma^{*}$) and RTT variation (RTTVAR, denoted as $V_{\gamma }$).
2. When the first RTT sample *γ* is obtained,
  (1) $$\gamma^{*}=\gamma ,$$
  (2) $$V_{\gamma }=\frac{\gamma }{2}.$$
3. When a new RTT is sampled, then
  (3) $$V_{\gamma }=\left(1-\beta \right)V_{\gamma }+\beta \left|\gamma^{*}-\gamma \right|,$$
  (4) $$\gamma^{*}=\left(1-\alpha \right)\gamma^{*}+\alpha \gamma ,$$
  where α=1/4 and β=1/8.
4. The RTO Ω is calculated by SRTT and RTTVAR as follows:
  (5) $$\Omega =\gamma^{*}+4V_{\gamma }.$$
5. When retransmission is triggered, exponential back-off is performed. Note that the specification suggests that RTO value is bounded within 1 and 60 s.

Note that RFC 6298 ignores the RTT samples of the retransmitted data, which follows Karn’s algorithm [9].

CoCoA, which is introduced as an Internet-Draft [10], enhances the congestion control mechanism for CoAP. Based on the calculation in RFC 6298, it defines two extra RTOs. One is called strong RTO (denoted as $\Omega_{strong}$) and $\Omega_{strong}=\gamma^{*}+4V_{\gamma }$. The other is weak RTO (denoted as $\Omega_{weak}$) and $\Omega_{weak}=\gamma^{*}+V_{\gamma }$. When there is no RTT sample, the RTO is set to 2 (i.e., Ω=2). The subsequent update of RTO occurs when an RTT is sampled. If the RTT is sampled from the first transmission of a message, the RTO is updated as

(6) $$\Omega =0.5\Omega +0.5\Omega_{strong}.$$

On the other hand, if the RTT is sampled from the retransmission of a message, the RTO is updated as

(7) $$\Omega =0.75\Omega +0.25\Omega_{weak}.$$

If the RTO is not updated over 30 s, the RTO is updated by

(8) Ω=(2+Ω)/2.

Another improvement is the back-off mechanism. In CoCoA, variable back-off factor (VBF, denoted as Δ) is decided based on the initial RTO as follows:

(9) $$\begin{array}{ccc} \Delta =3, & if & \Omega <1\;s,\\ \Delta =2, & if & 1\;s\leq \Omega \leq 3\;s,\\ \Delta =1, & if & \Omega >3\;s. \end{array}$$

Note that Δ is always equal to 2 in exponential back-off used by CoAP and RFC 6298.

When a message is sent, one of the two policies, persistent policy or Replacing-Retransmitted-Publication (RRP) policy, is chosen. In persistent policy, when a message is sent, the device drops following messages until it receives the corresponding ACK. While the number of retransmissions matches the maximum retransmissions number, the device does not wait anymore. RRP policy is like persistent policy except that if the message is retransmitted due to RTO timeout, the message can be replaced by a newly generated message. In previously described protocols, MQTT-SN uses persistent policy while CoAP, RFC 6298, and CoCoA use RRP.

We observe that neighboring devices would observe similar RTT (with the same server), and thus most dynamic RTO computation could be reduced by sharing the computed RTO values between devices. Based on those previous works, we propose a gateway-assisted mechanism to help devices get RTO earlier. Consequently, the number of retransmissions is reduced, which results in considerable power savings.

## 3. Gateway-Assisted CoAP (GaCoAP)

In this section, we introduce our main idea with the procedure of our scheme. Then, we briefly describe the RTO computation applied in our mechanism.

In our architecture, we consider a star topology, which means that each device connects to the gateway directly. The gateway is a transparent gateway between IoT devices and the server. In order to apply dynamic RTO, we consider that the gateway computes the RTO for the devices, connecting to it instead of computing by the devices themselves. Moreover, the devices can overhear the RTO values of other devices when they need to monitor the channel to ensure that the channel is idle before transmitting a packet.

The devices overhearing from this method may consume only a little extra power. However, the devices can obtain an appropriate RTO earlier by overhearing. Since the network status is similar for every device in the same wireless sensor network (WSN), the RTO value calculated by the gateway reveals the latest RTO of the WSN. If a device does not transmit data for a while, it can still update its RTO by overhearing. Having the latest RTO value reduces the data retransmission, which causes considerable power consumption. Note that, when no data is to be transmitted, repetitive overhearing may cause significant extra power consumption. Therefore, in our design, the devices overhear the RTO values only before they transmit packets.

When the device publishes the first message, the default RTO is set to 2. Once an RTT is measured by the device, RTO is recalculated for the next message. GaCoAP also adopts RRP policy when sending a message.

**Figure 3:**
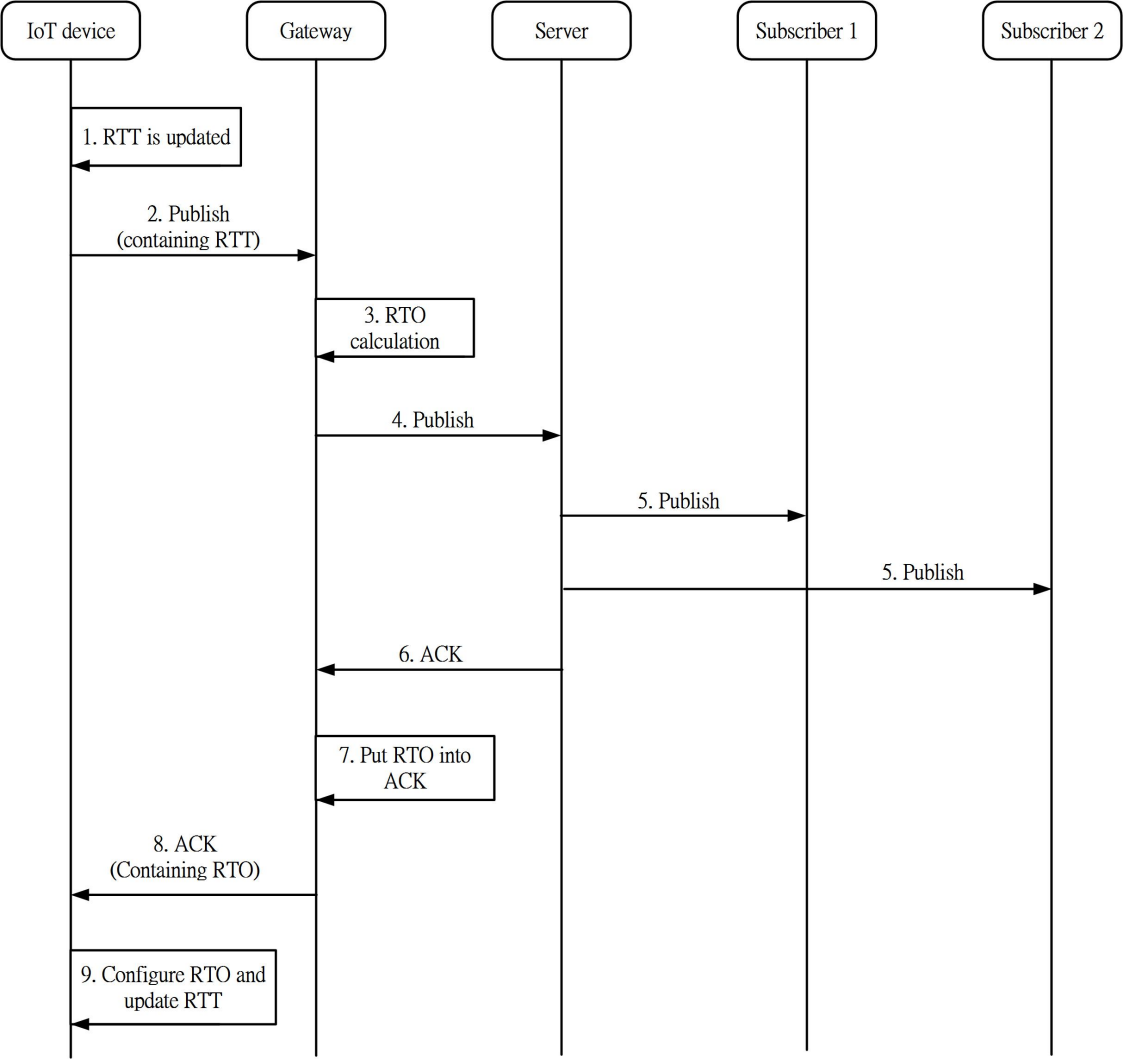
The procedure of our scheme.

Figure 3 shows the procedure of our scheme with the following steps:

1. The RTT is updated upon the receipt of the previous ACK and stored in the device for further use.
2. When the device publishes a new message, the RTT information is contained in the message and delivered to the gateway.
3. The gateway retrieves the RTT information to compute the RTO for the device and stores the RTO value in itself. After that, the gateway removes the RTT information from the message.
4. The gateway forwards the message to the server.
5. If some subscribers have registered the corresponding interest, the server forwards the message to them.
6. The server sends an ACK as a response to the device.
7. Before the gateway forwards the ACK to the device, it puts the RTO information into the ACK.
8. The gateway returns the ACK to the device. At the same time, the gateway broadcasts this RTO to other devices. While some devices are monitoring the downlink channel right at that time, they can receive the RTO information. If the coming RTO is larger than the one kept in it, it updates the RTO by the new one. If the devices are not monitoring, they just omit this information.
9. The device configures the RTO value and updates the RTT.

Finally, based on CoCoA, the RTOs are classified into strong RTOs and weak RTOs. The gateway retrieves the RTT information that contains the RTT value and the retransmission flag. If the retransmission flag is set up, the gateway adopts the weak RTO. Setting up the flag means that the RTT is obtained after at least one retransmission. On the other hand, the strong RTT is adopted if the ACK is received after the first transmission (i.e., there is no retransmission).

The variables SRTT and RTTVAR are calculated as Equations (1) and (2) if the RTT is measured during the first time, or Equations (3) and (4) are applied. Then, the RTO is updated by Equations (6) and (7), and the VBF in the back-off mechanism is as Equation (9). If the RTO value is not updated for 30 s, we use Equation (8) as the aging function.

In our design, we only have one GaCoAP relation, which is between the device and the server. In other words, there is no GaCoAP relation neither between the device and the gateway nor between the gateway and the server. The gateway is only in charge of RTO calculation and forwarding the messages between the device and the server. The RTT and the RTO occupy two extra bytes of the PUBLISH message and the ACK, respectively. Note that the RTT and the RTO are only delivered between the device and the gateway (and are thus unknown to the server).

## 4. Simulation Model

In this section, we describe our simulation model. Our simulation model for each method is implemented as an OMNet++ module [11]. IoT devices, gateways, and servers are three entities in our simulation. In the following experiments, the gateway connects to 30 IoT devices. For illustration purposes, we describe the simulation flow chart for IoT devices in GaCoAP. The simulation flow charts for gateways and servers are simpler, and are thus ignored in this paper.

**Figure 4:**
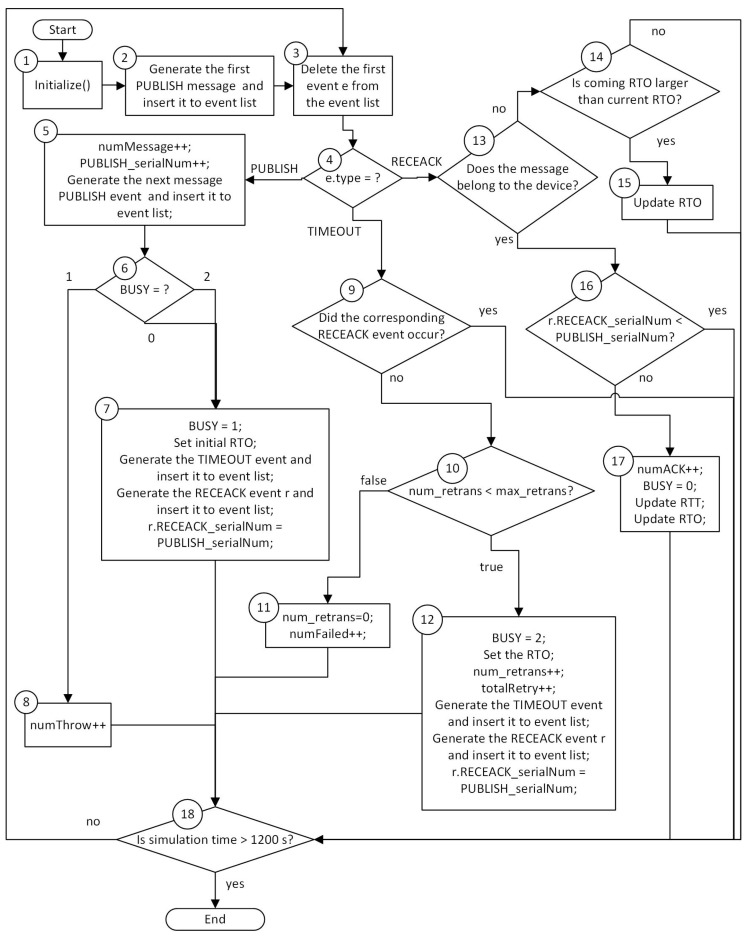
Simulation flow chart.

Figure 4 is the simulation flow chart for IoT devices in GaCoAP. There are several variables used in the simulation. **num_retrans** is the current number of retransmissions. **max_retrans** is the maximum number of retransmissions. **numMessage** is the number of messages published from the device. **numThrow** is the number of messages thrown out. **numFailed** is the number of messages that cannot be sent successfully. **totalRetry** is the total number of retransmissions. **numACK** is the number of ACKs successfully received by the device. **PUBLISH_serialNum** is the serial number of current PUBLISH messages. **RECEACK_serialNum** is the serial number of each received ACK message. **BUSY** parameter is used to indicate whether the device is waiting for ACK or not. If it is waiting for the ACK of the first transmitted message, BUSY is set to 1. If it is waiting for the ACK of the retransmitted message, BUSY is set to 2. Otherwise, BUSY is set to 0; **RETRANSMISSION** parameter is used to indicate whether this message is the retransmitted message or a newly generated message. If the message is a retransmitted message, RETRANSMISSION is set to true.

Steps in the simulation model are described as follows:

**Step** **1.** Devices initialize the setting. For example, numMessage is set to 0.
**Step** **2.** Generate the first PUBLISH message, which is treated as an event. Then, insert this event into the event list.
**Step** **3.** Delete the first event *e* from the event list. This event now needs to be processed.
**Step** **4.** Extract *e.type* to see what type this event is. If the type is PUBLISH, go to Step 5. If the type is TIMEOUT, go to Step 9. If the type is RECEACK, to to Step 13.
**Step** **5.** Parameters numMessage and PUBLISH_serialNum is increased by 1. Generate the next PUBLISH message and insert it to event list.
**Steps** **6–8.** Check BUSY status. If BUSY is 0, the device sends the message at Step 7. The condition BUSY = 2 means that the device is waiting for an ACK of retransmitted message. By applying RRP policy, the device stops waiting and sends the newly generated message. When the device sends the message, BUSY is set to 1. Set the initial RTO to the default value or the value calculated before. Generate the TIMEOUT event based on RTO and insert it into the event list, and also generate the RECEACK event and insert it into the event list. Set the RECEACK_serialNum of the RECEACK event to PUBLISH_serialNum. If BUSY is 1, numThrow is increased by 1, which means that this message is thrown out. Go to Step 18.
**Step** **9.** If the type is TIMEOUT, check whether the corresponding RECEACK event occurs or not. If the ACK is received, go to Step 18; otherwise, go to Step 10.
**Step** **10.** Check whether the device can retransmit the message. If num_retrans<max_retrans, go to Step 12 for retransmission; otherwise, go to Step 11.
**Step** **11.** The device has no quota to retransmit the message, so this message is not sent successfully. numFailed is increased by 1 and num_retrans is set to 0. Go to Step 18.
**Step** **12.** BUSY is set to 2 and the RTO is set up. num_retrans and totalRetry, are increased by 1. Generate the TIMEOUT and RECEACK events and insert them to the event list. Set the RECEACK_serialNum of the RECEACK event to PUBLISH_serialNum. Go to Step 18.
**Step** **13.** If the type is RECEACK, check whether this ACK belongs to the device itself. If this ACK does not belong to the device, go to Step 14; otherwise, go to Step 16.
**Steps** **14 and 15.** If the received RTO is larger than current RTO in device at Step 14, update the RTO with the newly received one, and then go to Step 18. Otherwise, just go to Step 18 directly.
**Step** **16.** Check whether RECEACK_serialNum of the received ACK is less than PUBLISH_serialNum. If the RECEACK_serialNum is less than PUBLISH_serialNum, just go to Step 18 because this ACK is not the one the device is waiting for now. Otherwise, go to Step 17.
**Step** **17.** The device receives the ACK message. Therefore, numACK is increased by 1, BUSY is set to 0, and the device calculates the RTT according to the received time of the ACK message. Finally, update RTO computed by the gateway and then go to Step 18.
**Step** **18.** This step checks the ending criterion. The criterion we use is whether the simulation is more than 1200 s or not. If the ending criterion is matched, end the program. Otherwise, go back to Step 3.

In our simulation, MQTT-SN [4], CoAP [5], CoCoA [10] and RFC 6298 [8] are also implemented based on relevant documents, and the details are ignored. Note that MQTT-SN uses persistent policy, and the other methods use RRP. In our experiments, we suppose that the fixed RTO value in MQTT-SN is set to 10 s. In CoAP, IoT devices choose a random number from 2 to 3 s as their initial RTO. Additionally, we suppose that the message generation intervals follow exponential distribution with a mean of five seconds. The maximum number of retransmissions in all methods is set to three.

## 5. Results

This section compares GaCoAP with MQTT-SN, CoAP, dynamic RTO in TCP (i.e., RFC 6298), and CoCoA under two different message loss rates (MLR): 0% and 10%. We introduce three output measures to investigate the performance. The first one is the average number of retransmissions in the device (denoted as *δ*). The second is the average message delivery ratio of the device (denoted as *ρ*). The last one is the average latency (denoted as *λ*), which means the round-trip time between the device and the server. We also investigate the impact of the number of devices connected to a gateway on *δ*. Finally, we discuss the power consumption in our method.

First of all, we introduce two functions to generate latency in our simulation. The latency is divided into two parts: one is the latency from device to gateway, and the other is the latency from the gateway to the server. The connection of the former is wireless while the latter is backbone. The latency varies with time, and the current one is related to the previous one. Based on the observation of RTT samples in [12,13], we use a saw-like function to formulate the latency in our simulation, and the slope of RTT model is 0.12. We assume that the saw-like function of the latency between the device and the gateway is as follows:

(10) $$latency=\left(2\mu \right)\left[\left(\frac{0.12}{2}\mu \right)t-\left\lfloor (\frac{0.12}{2}\mu )t\right\rfloor \right]+\left(2\mu +0.05\right),$$

where *μ* is the average value of the saw-like function and *t* denotes time. In our experiments, we consider four different values of *μ*, which are 1, 2.5, 5, and 10. On the other hand, we assume the saw-like function of the latency between gateway and server is as follows:

(11) $$latency=\left[\left(\frac{0.12}{2}\mu \right)t-\left\lfloor (\frac{0.12}{2}\mu )t\right\rfloor \right]+1.5.$$

**Figure 5:**
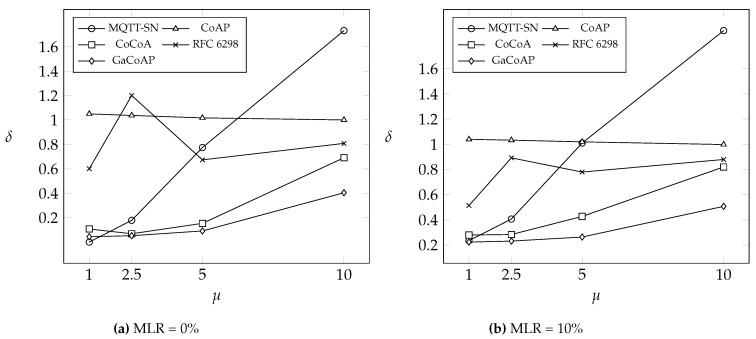
The average number of retransmissions *δ* under different *μ*.

Figure 5 compares the average number *δ* of retransmissions. As we know, if the RTO configuration is appropriate, the number of retransmissions should be small. MQTT-SN and CoAP use fixed RTO, which results in worse performance than the other three methods using dynamic RTO in many cases. This figure shows that *δ* in MQTT-SN significantly increases as *μ* increases, while *δ* in CoAP is insensitive to *μ*. This phenomenon is explained as follows. RTO in MQTT-SN is set to 10 s, so *δ* is small when *μ* is small. While *μ* becomes large, *δ* is getting worse. Take μ=5, for example, where the average one-way latency between device and gateway is 5.05 s. Obviously, RTO as 10 s is too small to be suitable for this situation. On the other hand, devices in CoAP choose RTO randomly from 2 to 3 s as their initial RTO. This RTO value is too small even though *μ* is set to 1. However, RTO is updated with an exponential back-off mechanism, such that *δ* becomes insensitive to *μ* in the observed range.

Compared to CoCoA and GaCoAP, RFC 6298 only takes RTT of first transmission into account and ignores the RTTs of retransmissions. This RTT information cannot reflect the entire network situation in real-time. As a result, the *δ* performance in RFC 6298 is worse than that in CoCoA and GaCoAP. In GaCoAP, RTO is not only based on the calculation in CoCoA but also updated by shared ACK message. Device in GaCoAP can obtain an appropriate RTO earlier so GaCoAP outperforms other methods, especially in bad network conditions. In the case that MLR =10% and μ=10, the devices adopting GaCoAP can save at most (1.902−0.507)/1.902=97.3% in terms of the number of retransmissions and (0.998−0.507)/0.998=49.1% at least.

**Figure 6:**
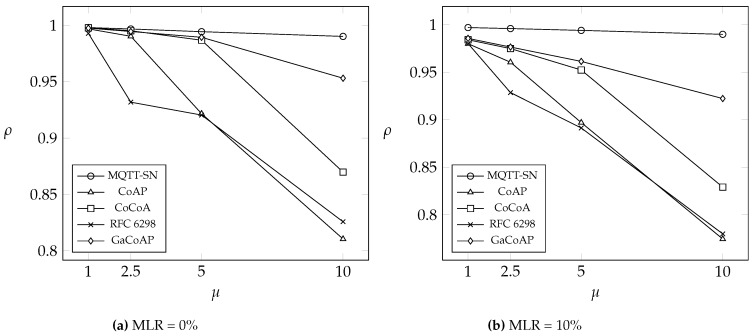
The average message delivery ratio *ρ* of device under different *μ*.

Figure 6 shows the effects of *μ* and MLR on the average message delivery ratio *ρ* (which means the ratio of the messages received by the server and the messages sent by the device). In this figure, MQTT-SN has the best performance because it uses the persistent policy (while others adopt RRP policy). Messages published by a device in MQTT-SN is not replaced by newly coming messages and the device always waits for the ACK of the current message it sends. Unfortunately, although *ρ* in MQTT-SN is large, the messages may be out-of-date. Since CoAP uses fixed RTO and adopts RRP policy, retransmission is triggered with higher probability, and then the current message is more likely to be replaced by a new message. On the other hand, the number of retransmissions in GaCoAP is small, so the message is hardly replaced. Even though working in the worst network conditions (i.e., MLR = 10% and μ=10), GaCoAP still has a 99.8% message delivery ratio, thus it improves the message delivery ratio to at most 0.998−0.774=22.4% among methods with RRP policy.

**Figure 7:**
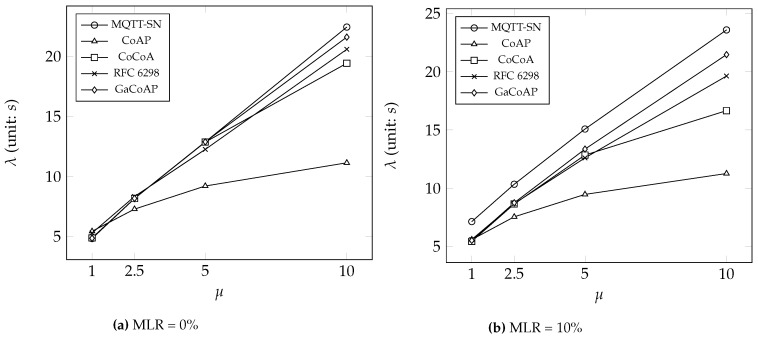
The average latency *λ* under different *μ*.

Figure 7 compares the average latency *λ* in each method. Overall, the latency in CoAP is the lowest because of frequent retransmissions. Once a message is lost, the device does not take a long time to wait for an ACK. Adopting an appropriate RTO can reduce the number of retransmissions, but this encounters larger latency. If the message is lost, the device may wait for more than a round-trip time and then trigger the message retransmission. Because RTO in GaCoAP is more suitable than other dynamic RTO methods, the latency in GaCoAP is a little bit larger. In the case that MLR = 10 % and μ=10, the devices adopting GaCoAP sacrifice at most 21 s − 11 s = 10 s latency. Note that 10 s latency is acceptible in most IoT applications. Finally, the latency in MQTT-SN is the largest due to its large and fixed RTO (i.e., 10 s).

**Figure 8:**
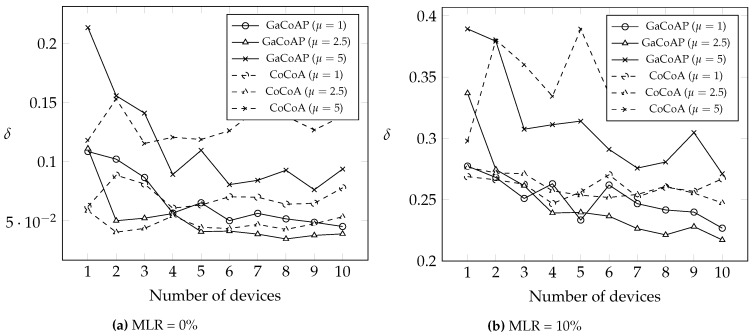
The average number *δ* of retransmissions under different numbers of devices.

To investigate the effect of moving RTO calculation from device to gateway, Figure 8 compares the average number *δ* of retransmissions in CoCoA and GaCoAP under different numbers of devices (connected to a gateway). Note that GaCoAP uses the same dynamic RTO calculation rules as that in CoCoA. After RTO calculation, the gateway in GaCoAP shares the RTO to other devices. The figure shows that, as long as there are more than five devices connected to a gateway, *δ* in GaCoAP is always smaller than that in CoCoA. When the number of devices is small, the effect of overhearing cannot play a role, and the device has to wait for RTO calculation by the gateway. In this situation, a device in CoCoA obtains suitable RTO quicker than that in GaCoAP. Nevertheless, a gateway certainly connects to more than five devices in practice. In fact, we extend our experiment up to 100 devices connected to a gateway, and the results are consistent.

Finally, we discuss the power consumption issue. Compared to other methods, devices in GaCoAP can save power owing to the reduction of retransmissions. If a device often triggers retransmission, it encounters higher transfer power consumption. In our experiments, the number of retransmissions in GaCoAP is the smallest, with only one exception (i.e., μ=1 and MLR = 0%). Assume that the packet size is 31 bytes, the size of Medium Access Control (MAC) ACK is 5 bytes, and the size of the application ACK is 12 bytes (this assumption follows the experiment setting in [14]). Based on Table 4 in [14], a device consumes 1182 ms·mA (summation of Id 3, Id 4, and Id 5) power when it transmits a packet. If the device needs to retransmit the packet, an extra 951 ms·mA (summation of Id 3 and Id 4) is required. For example, if the average number of retransmission is 0.1, the actual power consumption is 1182 + 951 × 0.1 = 1277.1 ms·mA. From Figure 5, the device in GaCoAP saves 1.902−0.507=1.395 retransmission times compared to MQTT-SN in the case that MLR = 10% and μ=10. As a result, each device can save 1840.185 ms·mA (951 ms·mA × 1.395) power for a packet at most. Even compared to CoCoA, the device in GaCoAP can still save 0.998−0.507=0.491 retransmission times. Each device saves at least 466.941 ms·mA (951 ms·mA × 0.491) power for a packet.

Indeed, compared to calculating the RTO by the device itself, sending two extra bytes for the RTT and receiving the RTO result in more power consumption. Compared to this consumption, the power savings of calculating the RTO is ignorable. According to [14], sending a packet over the air costs 31.5 ms·mA (see event 8). In our design, sending a packet that contains the RTT information occupies two extra bytes, which means that the device has to spend an additional 0.0645 ms (2 bytes/31 bytes). Therefore, the additional power consumption is 2.0318 ms·mA (0.0645 ms × 31.5 mA). On the other hand, the device updates the RTO in two ways. One is receiving its ACK with two extra bytes to carry the RTO. The other is receiving the ACK to get the RTO by overhearing. In [14], the required current for receiving MAC ACK or application ACK is 26.5 mA (see event 16). With the first way, the device has to receive two extra bytes, which costs an additional 1.70925 ms·mA (0.0645 ms × 26.5 mA). Using the second method, the device receives a whole 14 byte application ACK, which costs an additional 11.9677 ms·mA (0.4516 ms × 26.5 mA). Note that performing CSMA/CA observation also consumes energy, but it is common for all mechanisms (performing one observation consumes 1.325 ms·mA).

To put it briefly, a device adopting GaCoAP spends at most 13.677 ms·mA (per packet) to send the RTT and receive the RTO. However, this device can save at least 466.941 ms·mA per packet from reducing retransmissions. The power consumption mentioned above can be converted to other units for easy understanding. For example, a normal AAA battery (with capacity 1200 mAh) can be used to transmit 3,654,822 packets (1200×3,600,000/1182) if no retransmission occurs. If the average number of retransmission is 0.1, then only 3,382,664 packets (1200×3,600,000/1277.1) can be transmitted. In Figure 5, when MLR = 10% and μ=10, the energy required for a packet in GaCoAP is 1182+951×0.507+13.677=1677.834 ms·mA, while the energy required for a packet in MQTT-SN is 1182+951×1.902=2990.802 ms·mA. In this case, 2,574,748 packets can be transmitted in GaCoAP, while only 1,444,428 packets can be transmitted using a single AAA battery.

As a final remark, the network environment is simple in our experiment (a gateway connects to 30 devices), but it is more complex in the real sensor networks. From our simulation results, the GaCoAP mechanism outperforms the others, especially in worse conditions (i.e., large latency and high message loss rate).

## 6. Conclusions

In this paper, we proposed a gateway-assisted method for IoT devices to determine the RTO value of their wireless link. The main concept is transferring the calculation from devices to a gateway. The gateway dynamically calculates the RTO and then broadcasts the result to the devices connected to it. Using this method, the devices can save more power because they are able to obtain an appropriate RTO value earlier by overhearing. Then, we conducted experiments from four aspects to investigate the performance.

Compared to the other methods, the devices in GaCoAP can get dynamic RTO earlier, which results in much smaller retransmission times and larger message delivery ratio with some latency sacrifice. In the case that MLR = 10% and μ=10, the devices adopting GaCoAP can save at most 73.3% in terms of the number of retransmissions, and improve the message delivery ratio to at most 19% among methods with RRP policy. The devices only sacrifice up to 10 s (21 s−11 s) latency. Note that 10 s latency is ignorable in most IoT applications. The message delivery ratio in MQTT-SN is the best due to persistent policy; however, the number of retransmissions in MQTT-SN is the largest and the delivered message may be out-of-date. As long as the gateway connects to more than five devices, the GaCoAP mechanism outperforms all other mechanisms in terms of the number of retransmissions. Therefore, GaCoAP is better than other methods for IoT applications concerning energy usage.

Recently, several works have discussed applying Information Centric Networking (ICN) to IoT [15,16,17]. The IoT devices can benefit from this network paradigm in terms of power consumption, especially in multi-hop networks. Although we only consider the star topology in this paper, it is worth discussing how GaCoAP performs in ICN in the future. Another interesting work is applying lightweight application protocols on smartphones to improve performance of transmission. Since smartphones are embedded with various sensors and are widely used in modern society, it is important to involve smartphones in sensor networks. In [18], the performance of applying MQTT and CoAP to smartphones has been studied. Applying GaCoAP to smartphones brings more benefits (e.g., multiple sensors) as well as more challenges (e.g., mobility). We will also consider adopting GaCoAP on smartphones in future work.

## Figures and Tables

**Table 1:** Notations of Dynamic RTO in RFC 6298.

*γ*	round-trip time (RTT)
$\gamma^{*}$	smoothed round-trip time (SRTT)
$V_{\gamma }$	round-trip time variation (RTTVAR)
Ω	retransmission timeout (RTO)
